# Developing Partnerships to Reduce Disparities in Cancer Screening

**Published:** 2010-04-15

**Authors:** Erica S. Breslau, Phyllis W. Rochester, Debbie Saslow, Caroline E. Crocoll, Lenora E. Johnson, Cynthia A. Vinson

**Affiliations:** National Cancer Institute; Centers for Disease Control and Prevention, Atlanta, Georgia; American Cancer Society, Atlanta, Georgia; US Department of Agriculture, Washington, DC; National Cancer Institute, Bethesda, Maryland; National Cancer Institute, Bethesda, Maryland

## Abstract

**Background:**

Interventions in scientific settings to improve the well-being of women who are not regularly screened for cancer have failed. Consequently, community-based prevention and control efforts are needed.

**Community Context:**

From 2003 through 2007, three federal agencies and 1 nongovernmental agency collaborated with county-level public health counterparts from 6 states to address screening disparities in cervical and breast cancer in counties with the highest prevalence. This case study describes lessons learned from Team Up, a model pilot program.

**Methods:**

We conducted a descriptive qualitative case study including 5 Southern states and 1 Midwestern state: Alabama, Georgia, Kentucky, Missouri, South Carolina, and Tennessee. The 6 states underwent a 5-step process to adopt, adapt, and implement 1 of 3 evidence-based interventions designed for cervical and breast cancer screening.

**Outcome:**

The 6 participating states had various levels of success. Participating states formed and sustained viable interorganizational public health partnerships throughout the pilot program and beyond.

**Interpretation:**

Although this innovative pilot faced many difficulties, participants overcame substantial obstacles and produced many key accomplishments. Team Up brought together 2 challenging public health strategies: the translation of evidence-based approaches to communities and populations, and partnerships among diverse people and organizations. Case study results suggest that using a mix of approaches can promote the transference of evidence from research into practice through local, regional, and national partnerships.

## Background

As the Institute of Medicine reported in 2006, the health of racial and ethnic minorities, poor people, and other disadvantaged groups in the United States is worse than the health of the overall population (1). National health status reviews, including *Healthy People 2010*, have given a high priority to these associated excess illnesses and deaths, termed "health disparities" (2,3). Researchers have developed a blend of population-based strategies to improve the well-being of women who are not regularly screened for cervical and breast cancer; these strategies include implementing evidence-based practice guidelines and collaborating between public and private partners (4-7).

For many years, researchers assumed that implementing an intervention deemed effective in a research context into practice settings was not difficult (4). However, interventions developed in scientific settings to address cancer health disparities have failed because they are not fully understood, are not fully integrated into routine practice, are underused, or do not draw on the collaboration of practitioners working across different organizations and geographic regions (5). For community-based cancer prevention and control endeavors, creative strategies are necessary to address public health problems (8).

## Community Context

From 2003 through 2007, 3 federal agencies — the Centers for Disease Control and Prevention (CDC), the National Cancer Institute (NCI), and the US Department of Agriculture (USDA) — and 1 nonprofit national agency — the American Cancer Society (ACS) — partnered to conduct a pilot case study called Team Up. In addition to national partners, the pilot comprised state and county public health practitioners from 6 states: Alabama, Georgia, Kentucky, Missouri, South Carolina, and Tennessee. These states were chosen because they contained counties with the highest death rates and the lowest screening rates for cervical and breast cancer in the United States (9,10).

The objective of Team Up was to encourage regional public health programs to use cancer control approaches that are evidence-based to reach underserved groups. Typically, different agencies and organizations that target specific cancers undertake cancer prevention and control efforts, lacking coordination and collaboration (11). Such efforts also tend to use interventions that are not evidence-based. We saw an opportunity for regional programs and organizations to build capacity and improve health outcomes by partnering with national agencies and organizations. Communities with low screening rates could benefit from research and subsequent translation of interventions into evidence-based practice. Furthermore, multilevel partnerships — alliances formed between federal, regional, state, and community groups for a common purpose — needed to be studied as a conduit for using evidence-based approaches to encourage behavior change. Promoting evidence-based research is a federal priority, so the Team Up case study is described from the federal perspective.

## Methods

Team Up had 5 phases: 1) development and planning, 2) partnership formation and building, 3) capacity building, 4) implementation of evidence-based strategies, and 5) evaluation.

**Figure 1:**
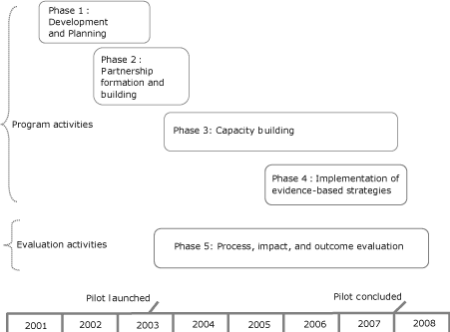
Team Up: Pilot program (2001-2007) and evaluation (2003-2008) phases.

### Development and planning (phase 1: November 2001-June 2003)

Organization and coalition literature identifies a sequence of phases through which organizations or pilot programs need to move as they establish themselves to meet goals (12). By design, the development and planning of Team Up took time and included a series of diagnostic events that occurred before the official pilot launch in 2003.

To build the foundation for partnerships, we conducted a participatory needs assessment to develop a structured approach to potential programmatic and evaluation activities. Programmatic activities included 2 components: developing a concept map to understand outcome domains that needed to be addressed (13) and building a logic model to depict the sequential and causal relationships among outcome constructs identified in the concept map (14). The results created a bridge between the diverse cultures of participants from the research-funding agency (NCI) and service delivery-funding agencies (ACS, CDC, USDA) to build a partnership based on 3 core goals: 1) to reach women who are rarely or never screened; 2) to use evidence-based interventions as the approach; and 3) to forge partnerships as collaborative relationships.

### Partnership formation and building (phase 2: June 2002-December 2003)

Partnerships between institutions that conduct research and those that deliver health care and social services can help bridge the gap between knowledge and practice (15-17). Even if partners have worked together in another capacity, new partnerships can be inconsistent because of their unique mission or partner composition (6,11).

**Figure 2:**
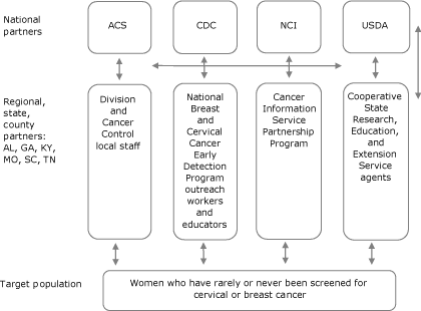
Structural framework of Team Up partnership. Abbreviations: ACS, American Cancer Society; AL, Alabama; CDC, Centers for Disease Control and Prevention; GA, Georgia; KY, Kentucky; MO, Missouri; NCI, National Cancer Institute; SC, South Carolina; TN, Tennessee; USDA, United States Department of Agriculture.

The 4 national partners (ACS, CDC, NCI, and USDA) provided initial support for Team Up and encouraged wide-reaching collaboration between preexisting health program infrastructures that regional, state, and county-level partners could access. Sources of partnership infrastructure included ACS's regional offices and Division of Cancer Control (DCC) staff, CDC's state and county-level National cervical and breast Cancer Early Detection Program (NBCCEDP), NCI's Cancer Information Service (CIS), and USDA's Cooperative State Research, Education, and Extension Service (CRESS) agents (Figure 2). We selected USDA agents because of their established educational programs in communities of interest and their access to underserved women. Regional, state, and county-level partners (CIS, CRESS, DCC, and NBCCEDP) were known as "state partners." Throughout the life of the pilot program, national partners made efforts to engage state partners whenever possible to use their access to unscreened women.

### Capacity building (phase 3: July 2003-September 2007)

In 2003 Team Up developed a series of capacity-building activities to ensure that state partners had adequate skills and training to select and deliver complex evidence-based interventions appropriate for target populations. Capacity building provided tailored technical assistance for implementation activities. We originally planned these activities as a single kickoff meeting, but Team Up state partners required ongoing mentoring for the life of the pilot program. In all, we provided 9 capacity-building activities:

1. Kickoff meeting (July 2003). The national partners developed a kickoff training program for state partners. This launch of the Team Up pilot offered training and follow-up activities designed 1) to develop, strengthen, and support regional, state, and local-level partnerships; 2) to identify, access, adapt, and implement evidence-based approaches for use in high-mortality regions; and 3) to identify and encourage women who have rarely or never been screened for cervical or breast cancer to be screened. Each state developed an action agenda to guide its next steps. 
2. Web forum (June 2004-October 2006). States requested a Web-based medium for sharing common materials, including presentations and formal documents from the national partners that they could use locally, information about planning the implementation, and training announcements. CDC led the Web forum, which encouraged communication through regular live chats among state partners. 
3. Newsletters (October 2004-April 2007). The 11 issues of the Team Up newsletter from the national partners were the most frequent formal communication used 1) to share broad technical assistance needs for education, 2) to highlight partnership success with specific states, 3) to document the implementation progress of Team Up, and 4) to provide general communication with states during the pilot. 
4. Coaches (October 2004-December 2007). Because progress after the kickoff meeting was slow, the states asked for additional assistance to clarify technical issues, strengthen relationships, and provide assistance on how best to accomplish the multifaceted Team Up objectives. Two coaches worked one-on-one with state partnerships to build capacity and provide technical assistance. 
5. PATH visits (April 2005-May 2005). We used a combination of tailored teleconferences and in-person visits to develop a personalized approach. Partnership Assistance and Technical Help (PATH) visits allowed state partnerships to refine technical assistance plans and identify priority action steps to accomplish Team Up goals. 
6. Regional meetings (June 2005-August 2005). National and state partners attended 1.5-day regional meetings designed to foster a common understanding of Team Up goals, objectives, concepts, methods, and timelines. The 2 meetings provided a venue for states to share general technical strategies and convey concerns. 
7. Webinars (October 2005-April 2006). Web-based seminars (webinars) were mini-conferences initiated by the national partners on different topics identified by state partners. The 3 webinars addressed specific technical assistance needs, facilitated live collaborative exchange of information between state and national partners, and hosted guest lectures and question-and-answer sessions with subject-matter experts. 
8. Retreats (June 2006-January 2007). Retreats focused on operationalizing strategic plans and implementing and evaluating action steps. Although we invited all 6 states to participate in the 2-day retreats conducted by ACS, only 3 that were at a developmental phase participated. 
9. National meetings (August 2006 and June 2007). Two national meetings provided an opportunity for national and state partners to network, share progress and experiences, and receive training or technical assistance from experts.

### Implementation of evidence-based interventions (phase 4: July 2005-December 2007)

In the implementation phase, state partners translated research into practice through the delivery of evidence-based approaches to reach rarely or never screened women. All 6 states moved through a sequence of 5 core steps as they became familiar with new terminology and activities. Step 1 involved preparatory steps during which state staff conducted diagnostic needs assessments, collected surveillance data, and convened planning meetings. In many instances, these smaller planning groups became the nuclei for larger state initiatives with partners who would eventually deliver the intervention. In step 2, the state partners assessed interventions to determine if they were appropriate for their target populations. Interventions deemed appropriate were adopted. In step 3, adaptation involved fitting the specific intervention to the real world or field settings. Step 4 involved implementation and included training staff to deliver the intervention to women among whom rates of cancer screening were poor. In step 5, the state partners evaluated previous activities.

### Evaluation (phase 5: July 2003-April 2008)

**Figure 3:**
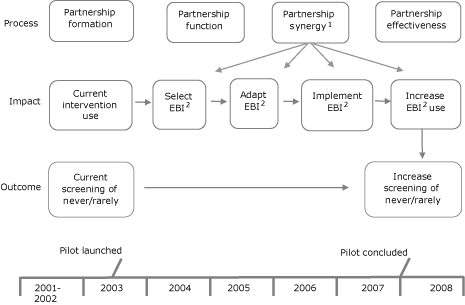
Team Up evaluation organizational framework. Abbreviation: EBI, evidence-based intervention. 1. Partnership synergy is a collaborative process that enables a group of people and organizations to combine complementary knowledge, skills, and resources to accomplish more as a group than as individuals (Lasker and Weiss, 2003). The Lasker and Weiss Partnership Self-Assessment Tool identifies a partnership's strengths and weaknesses in areas known to be related to synergy: leadership, efficiency, administration and management, and sufficiency of resources. Response categories are based on 5-point Likert scales (extremely well [5] to not at all well [1]; excellent [5] to poor [1]; all of what it needs [5] to none of what it needs [1]). Overall synergy results are based on a compilation of definitive questions with the resulting categorical scores: Danger Zone (1.0-2.9) requires a lot of improvement; Work Zone (3.0-3.9) requires effort to maximize the partnership's collaborative potential; Headway Zone (4.0-4.5) encourages greater potential to progress further; and Target Zone (4.6-5.0) requires focus to maintain a synergistic partnership (http://partnershiptool.net/). 2. EBI: Evidence-based intervention. The term "evidence-based intervention" refers to an intervention that has been tested through randomly controlled experiments with efficacious results that have been published in peer-reviewed journals (http://www.aoa.gov/doingbus/fundopp/announcements/2008/ADDGS_Evidence_Based_FAQ.doc).

The organizational framework used in the evaluation planning and design (Figure 3) shows the relationship of the pilot program's programmatic elements to the relevant short-term (eg, formation of partnerships) and midterm (eg, knowledge of and application of evidence-based methods) outcomes.

**Table:** 

This figure is titled “Team Up evaluation organizational framework.” There are 3 rows headed, from top to bottom, *process*, *impact*, and *outcome*. Items in the first row are *partnership formation, partnership function, partnership synergy,* and *partnership effectiveness*. Items in the second row are *current intervention use, select EBI, adapt EBI*, and *implement EBI use*. The third row has 2 components: to the left, *current screening of never/rarely*, and on the far right, *increase screening of never/rarely*. A timeline runs horizontally below, showing that the pilot was launched in the first quarter of 2003 and concluded at the end of 2007. Arrows in each row point from the first item in each row to the next, until the last item. Under the item *partnership synergy*, 4 arrows point to items in the next row: *select EBI, adapt EBI*, and *implement EBI use*. EBI is an abbreviation for *evidence-based intervention*.
The legend defines *partnership synergy* and *evidence-based intervention*. The legend says:
Partnership synergy is a collaborative process that enables a group of people and organizations to combine complementary knowledge, skills, and resources to accomplish more as a group than as individuals (Lasker and Weiss, 2003). The Lasker and Weiss Partnership Self-Assessment Tool identifies a partnership’s strengths and weaknesses in areas known to be related to synergy: leadership, efficiency, administration and management, and sufficiency of resources. Response categories are based on 5-point Likert scales (extremely well [5] to not at all well [1]; excellent [5] to poor [1]; all of what it needs [5] to none of what it needs [1]). Overall synergy results are based on a compilation of definitive questions with the resulting categorical scores: Danger Zone (1.0-2.9) requires a lot of improvement; Work Zone (3.0-3.9) requires effort to maximize the partnership’s collaborative potential; Headway Zone (4.0-4.5) encourages greater potential to progress further; and Target Zone (4.6-5.0) requires focus to maintain a synergistic partnership (http://partnershiptool.net/). EBI: Evidence-based intervention. The term “evidence-based intervention” refers to an intervention that has been tested through randomly controlled experiments with efficacious results that have been published in peer-reviewed journals (http://www.aoa.gov/doingbus/fundopp/announcements/2008/ADDGS_Evidence_Based_FAQ.doc).

The purpose of the 3-part theoretically guided evaluation was to determine whether Team Up achieved its goals. Part 1, the process evaluation, assessed the success of the state partners in building a synergistic partnership that resulted in an intersection of common knowledge, skills, and consensus (6). Part 2, the impact evaluation, measured attributes that influenced adoption, adaption, and implementation of evidence-based interventions. Part 3, the outcome evaluation, assessed whether state partners influenced short-term screening rates through the interventions.

## Outcome

### Partnerships

On the basis of data collected throughout the pilot program, all Team Up states developed sustained public health partnerships (18). The states' success in moving from a team of people to a viable partnership was measured by the extent to which individual and organization perspectives, nonmaterial resources, and skills of participating people and organizations contributed to and strengthened partnerships as a whole (6,18). By the end of the pilot, states with strong leaders obtained external funding, had dedicated staff, and collaborated across partners. In comparison, states with less robust leadership encountered interorganizational challenges that required considerable partner efforts to reconcile (18). This conflict was a consequence of partners' having opposing goals and different problem-solving styles, agendas, and resource capacities.

### Evidence-based intervention

Six states implemented an evidence-based screening intervention for breast or cervical cancer. The evidence-based interventions were selected, and specific components were implemented (Table). To select interventions, states consulted peer-reviewed publications, professional organizations, and *The Guide to Community Preventive Services: What Works to Promote Health?* (http://www.thecommunityguide.org/) and linked to interventions via a Web portal (Cancer Control PLANET [Plan, Link, Act, Network with Evidence-based Tools] http://www.cancercontrolplanet.com/). From the cancer control interventions available, combinations of 3 strategies were used.

## Interpretation

Team Up offered promising strategies for accelerating the delivery of research-tested approaches into practice (19). Through partnerships, the pilot generated collaboration among diverse people and organizations enhanced understanding of implementation concepts and strategies (5). However, the resultant Team Up pilot also encountered many difficulties. Launching the pilot revealed a need for technical assistance in the areas of partnership maintenance and successful implementation. As a result, various strategies to enhance relationships were considered together with practical methods to contextualize the implementation of evidence into practice. After Team Up launched, national partners recognized the need to offer extensive capacity-building to assist state partners, an investment not initially realized. Further, since the evolution of Team Up, implementation science has become more predominant within public health (20). Consequently, the methods described here provide a useful context for other public health projects seeking to apply evidence-based strategies to decrease gaps in screening for cervical and breast cancer.

Promoting and encouraging the transfer of evidence from research into practice required a mix of methodologic approaches and a discourse between those moving the evidence from one environment to another. Although the results of Team Up were highly variable, successes were realized, and many lessons emerged.

### Expanding the local partner base

Because each partner brought a unique perspective to Team Up state partnerships, states without full involvement from all partners had difficulty achieving Team Up's goals. As in any collaborative initiative, reconciling these different partnerships required identifying and integrating complementary nonfinancial resources. Nonetheless, the partnerships worked to overcome their respective challenges. The collective commitment to the Team Up pilot goals and a respect for each other kept the 6 partnerships from separating.

Continuity of membership from initiation throughout the life cycle of the partnership is vital when interorganizational partners are involved. People and organizations that have experience working in the target communities come from diverse constituencies. These valuable partners typically know what is feasible and realistic, thus improving "buy-in" and participation during the delivery of the intervention (ie, the implementation phase). Involving and training people with backgrounds similar to those of the target population from the beginning can increase ownership, communication, and commitment and reduce turnover (11,17). Providers of public health interventions in underserved or minority populations also may require ongoing training in cultural competency (7,21). In addition, key leaders at all levels juggled competing priorities, nonfinancial resources, and their diverse sectors.

### Understanding the implementation context

Until recently, the availability of evidence-based screening interventions specifically designed for underscreened populations was limited. Commonly, staff needed to be trained to identify appropriate interventions and how to use the Cancer Control PLANET and *The Guide to Community Preventive Services*. Both resources offered examples of interventions for different populations and contextual environments. Access to intervention components through contact with researchers diminished the research-to-practice gap (22).

When practitioners considered what to adopt, state partnerships did not always agree on what counted as evidence and under what circumstances. For example, interventions that did not contain the entire "suite" from the original research intervention may not maximize effectiveness in the new practice environment, a fact not measured in this pilot program. Furthermore, because of the demographic and geographic diversity of women within counties, research interventions were adapted at multiple levels of the partnership and continually evolved. Team Up found it essential to systematically track the adaptation progress throughout the implementation process. State partners repeatedly expressed the idea that "one size does not fit all," and this belief translated into innovative county- and community-level adaptation, something that exceeded the state partnership initial plans.

### Adapting evidence-based interventions

No universal recommended process or set of established best practices exists for the adaptation of evidence-based interventions to populations, conditions, and environments different from those in the original research (23). Although adaptation is necessary to make the intervention more relevant for a new target population, the more intervention components were altered, the less fidelity to the original intervention remained. On the other hand, the process enabled those who were adopting the evidence-based intervention to gain ownership of the new intervention. A deeper question is whether we can or even need to develop specific early detection programs for every combination of language, culture, geographic location, and racial/ethnic subgroup (21). Even though the implementation context is vital, more important is the knowledge of how much an intervention can be altered before it becomes completely different (22).

### Sustaining the pilot model

Collaborations formed early in the partnership can be sustained beyond the pilot and can overcome obstacles in successful and creative ways. Synergy is a key measure of partnership success (6,18). In Team Up, synergy appeared to increase over time. However, in 1 state where leadership changed, the partnership synergy seriously dissipated for a time. In general, an indicator of Team Up's success is the strong leadership and management structures that enhanced collaboration at the local level. Several states plan to sustain the Team Up model with other cancer control efforts.

Team Up had certain limitations. First, even though the Team Up multilevel partnerships were integral to programmatic goals, each state partnership functioned as a distinct unit with strong ties to both the local communities they operated in and to particular national organizations. Generalizing these methods to other multilevel partnerships interested in focusing on cancer control and prevention is encouraged; however, this task may be difficult because of the populations that state partnerships chose to reach. Team Up was a case study, and one cannot generalize from a single case (24). Second, evaluation outcomes need to be accompanied by an understanding of why or how a specific strategy did or did not work (25). Finally, future programs should consider building in a cost assessment associated with implementing such a multiyear program in several states.

As a case study, Team Up allowed us to examine a complex approach to address cancer screening disparities and to consider methods for translating scientific knowledge into practice. Although the field of health care disparities is firmly established, public health strives to find a practical blend of strategies and interventions that effectively work to reduce these disparities. This case study suggested that a combination of approaches should be tried before moving to a larger-scale study.

## Figures and Tables

**Table.** Intervention Activities for Team Up, 2003-2007

State	Intervention^a^	Components	Year
Alabama	Forsyth County Cancer Screening Program	Media campaign, educational classes,^b^ 1-on-1 sessions	2005
Georgia	Forsyth County Cancer Screening Program,^b^ Filipino American Women's Health Project	Educational classes	2005
Kentucky	Forsyth County Cancer Screening Program	Media campaign, educational classes,^b^ 1-on-1 sessions, educational games to teach exam skills, distribution of literature in the waiting room	2004
Missouri	Breast Cancer Screening Among Nonadherent Women	Tailored telephone counseling, tailored print communications	2005
South Carolina	Forsyth County Cancer Screening Program	Educational classes,^b^ in-service and primary care conference training for providers	2005
Tennessee	Forsyth County Cancer Screening Program	Media campaigns, educational classes,^b^ 1-on-1 sessions, direct mail, community events, in-service and primary care conference trainings for providers, distribution of literature, 1-on-1 counseling sessions, and personalized follow-up letters for women with abnormal test results	2005

a Before Team Up, states reported using 47 different intervention strategies, most of which lacked evidence-based approaches. Interventions comprised community awareness, education programs, church events, lay health advisor, health fairs, and media campaigns. All the interventions selected in Team Up were shown to be efficacious in increasing screening for cervical and breast cancer or both in the original population.

b Five states included educational classes with a church as either the recruitment or intervention site.
